# The RNA editing landscape in acute myeloid leukemia reveals associations with disease mutations and clinical outcome

**DOI:** 10.1016/j.isci.2022.105622

**Published:** 2022-11-17

**Authors:** Eshwar Meduri, Charles Breeze, Ludovica Marando, Simon E. Richardson, Brian J.P. Huntly

**Affiliations:** 1 Wellcome - MRC Cambridge Stem Cell Institute, Cambridge, UK; 2 Department of Haematology, University of Cambridge, Cambridge, UK; 3 Cambridge University Hospitals, Cambridge, UK; 4 UCL Cancer Institute, University College London, London WC1E 6BT, UK

**Keywords:** Cancer systems biology, Cancer, Transcriptomics

## Abstract

Several studies have documented aberrant RNA editing patterns across multiple tumors across large patient cohorts from The Cancer Genome Atlas (TCGA). However, studies on understanding the role of RNA editing in acute myeloid leukemia (AML) have been limited to smaller sample sizes. Using high throughput transcriptomic data from the TCGA, we demonstrated higher levels of editing as a predictor of poor outcome within the AML patient samples. Moreover, differential editing patterns were observed across individual AML genotypes. We also could demonstrate a negative association between the degree of editing and mRNA abundance for some transcripts, identifying the potential regulatory potential of RNA-editing in altering gene expression in AML. Further edQTL analysis suggests potential *cis-regulatory* mechanisms in RNA editing variation. Our work suggests a functional and regulatory role of RNA editing in the pathogenesis of AML and we extended our analysis to gain insight into the factors influencing altered levels of editing.

## Highlights

• Increased levels of RNA editing are a predictor of poor survival in AMLs
• Differential levels of RNA editing are observed among AML genotypes
• mRNA abundance negatively correlates with the degree of editing of some transcripts
• RNA editing variation is partly explained by cis-regulatory mechanisms

## Graphical abstract

**Figure:**
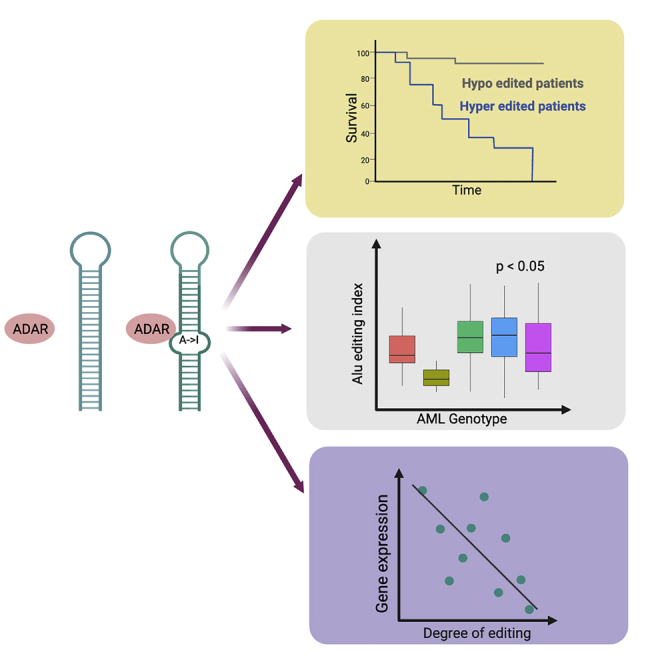

## Introduction

Acute Myeloid Leukemia (AML) is an aggressive, often fatal hematological malignancy, where the normally exquisitely regulated processes of self-renewal, proliferation, and differentiation within the hematopoeitic system are aberrantly coordinated.^1^ Transcriptional alterations have been shown to be a cardinal feature of AML, and can be explained, at least in part, by recurrent mutations of transcription factors and epigenetic regulators.^2^^,^^3^ Mutations of members of the RNA-splicing machinery are also common and more recently RNA-modifications, such as N^6^-methyladenosine (m6A), have also been shown to be of mechanistic importance. Editing of RNA, alternatively termed as “RNA-DNA differences” (and hereafter RDDs), is another such putative “epitranscriptomic” modification that may alter gene expression and cellular phenotype, without permanently altering the DNA sequence. Although not the only modification, the major editing event is the alteration of Adenosine (A) to Inosine (I), which in turn is read as Guanine (G) by the translational machinery. This activity is predominantly catalyzed by the ADAR enzymes.^4^

Although RNA editing was originally described over 30 years ago, only recently, with the advent of deep sequencing technologies, have millions of RDDs been documented across healthy individuals and several cancers.^5^^,^^6^^,^^7^^,^^8^^,^^9^^,^^10^^,^^11^ RDDs predominantly occur in non-protein-coding DNA regions, where they have been proposed to alter the recognition of transcripts by the RNAi machinery and/or to alter RNA splicing. A small proportion of RDDs lie in the coding regions and may alter protein sequence. To estimate the clinical relevance of RNA editing in cancers, several studies have compared tumors with tissue-matched controls, documenting site-specific and global (per-sample measure) differential patterns of RNA editing. Site-specific analyses have revealed that editing levels remain similar to matched control samples at the majority of sites. However, some sites demonstrate hypo/hyper editing patterns. Increased levels of RNA editing in coding sequences lead to demonstrated alterations of the encoded protein. For example, *AZIN1* (S367G) in Hepatocellular carcinoma and *RHOQ* (N136S) in Colorectal cancer, have been shown to be associated with tumorigenesis. Similar trends are observed for global measures of RNA editing, where the majority of cancers have been reported to have elevated levels of editing. Furthermore, it has been shown for most tumors that increased levels of editing are associated with poor survival outcomes.^9^^,^^11^ Of all the cancers studied, reduced levels of editing are reported only in Glioblastomas (GBM), where, interestingly, RNA editing acts in a gender-specific manner on patient outcomes, with high levels of editing being favorable in males,^12^ although the basis for this remains unknown.

In an earlier study across AML patient samples, a hyper-edited event at an intronic branchpoint of the *PTPN6* (*SHP1*) transcript was linked to disease progression.^13^ In addition, analyses of ADAR expression and activity have revealed that increased editing activity occurs during myeloid cell differentiation.^14^ However, these studies were performed on smaller datasets. High throughput sequencing data of several cancers from the TCGA have been available for several years. RNA editing profiles across these samples were studied by several research groups.^9^^,^^11^^,^^12^^,^^15^ However, the role of RNA editing in AMLs from a large study, such as the TCGA cohort remains largely unexplored. Here we took the advantage of high throughput transcriptomic dataset from The Cancer Genome Atlas (TCGA) study for 151 AML patient samples to explore to what extent RNA editing may play a role in AML. We suggest a functional role for RNA editing in AML, by associating it with survival outcomes and with the mutational status of patient samples. These results are further validated by a recently published publicly available dataset from the BeatAML consortium.^16^ Furthermore, we suggest a regulatory potential for RNA editing by associating it with mRNA abundance. Finally, in an attempt to understand the factors influencing differential editing levels, we perform cis-edQTL analysis with the imputed genotypes.

## Results

### RNA editing in acute myeloid leukemia

**Figure 1:**
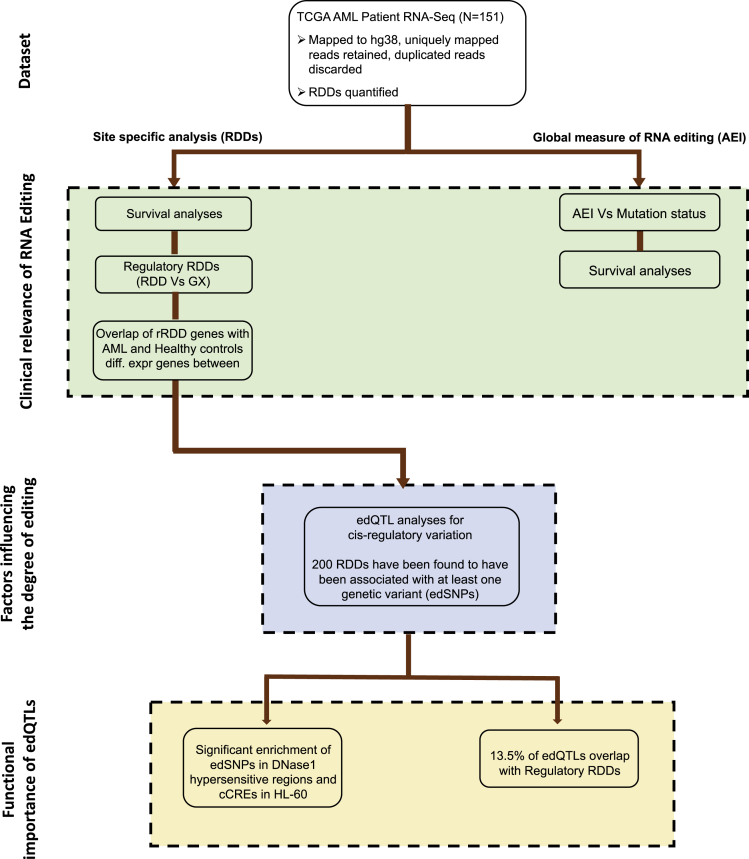
Study design Results are presented in four different sections as shown on the left.

**Figure 2:**
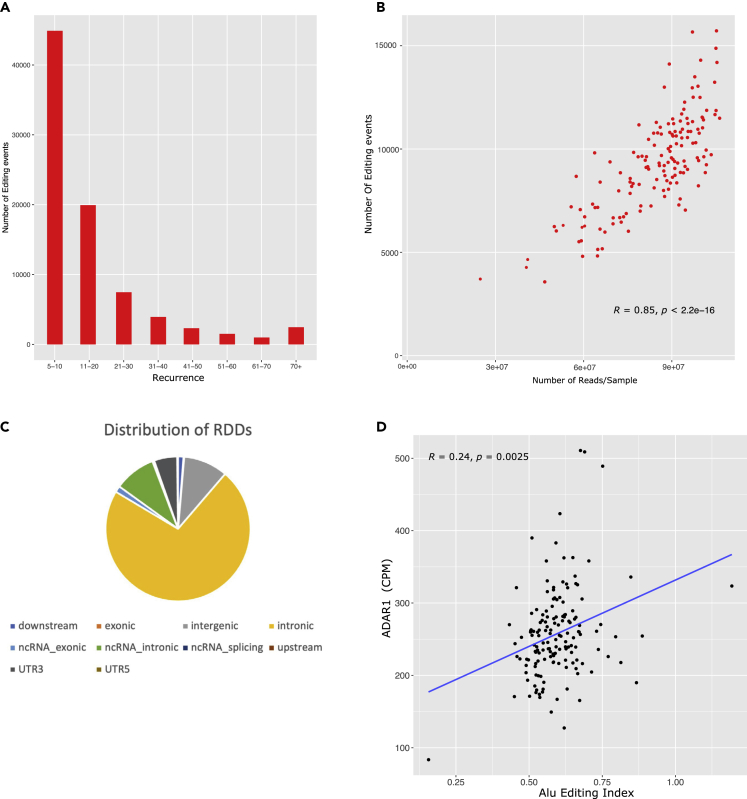
Distribution of RDDs and association between AEI and ADAR1 (A) TCGA AML RDDs were categorized into groups based on their recurrence. Intervals on x axis represent the number of samples in each group. Number of RDDs is shown on the y axis. (B) Correlation between number of RDDs (*y axis*) in each sample and number of reads per sample (*x axis*). Spearman correlation and p *value* are shown. (C) Genomic locations of RDDs quantified in the AML dataset. As described in Annovar, “upstream” and “downstream” are defined as within 1-kb from the transcription start and end site, respectively. (D) Correlation plot between AEI and ADAR1 gene expression (CPM). Each dot represents an AML patient sample. The straight blue line represents the linear relationship between the AEI and ADAR1 expression.

A schematic of the analytic pipeline for the study is shown in Figure 1. To first determine if RNA editing was altered in Acute Myeloid Leukemia, we studied the TCGA dataset,^17^ where 151 RNA-seq samples were available for analysis. This patient cohort consists of 82 males and 69 females with a median age of 56 years. We could demonstrate that the majority of the individual RDDs are recurrent in only a few samples (Figure 2A). Therefore, to avoid false positives and to focus on common events, we report the RDDs that were recurrent in at least five individuals. Of note, marked heterogeneity was seen in the number of RDDs between AML individual patient samples, with a range from 3,572 to 15,723 editing events demonstrated per sample (average 9230 RDDs) (Figure 2B). In the AML patient samples, we identified 78,035 and 5503 edited sites in the Alu and Non-Alu regions respectively (Table S1). However, as has been shown in previous studies, we found the number of editing sites to be strongly correlated to the sequencing depth (rho = 0.85, p value < 2.2e-16) (Figure 2B). RDDs were predominantly located in the non-protein-coding regions, with only 123 events across 72 genes located in exonic regions. Of interest, however, double the number of non-synonymous substitutions was observed in comparison to synonymous substitutions at these sites (Table S1). In a recent study across several cancers, non-synonymous editing events were functionally characterized, based on survival probabilities and differential editing levels across tumor subtypes.^9^ Whilst we note that the quantification of RNA editing is a function of sequencing depth and cohort size, we could still replicate the presence of 16 of their 35 non-synonymous editing events in our AML dataset (Table S1). Of note, from the above 72 genes, *HAUS3, PUS1, SDHAF2, SRP9,* and *ZNHIT3* were determined to be predicted vulnerabilities in AML by our previous genome-wide CRISPR -dropout screen in AML cell lines.^18^ RDDs were also found in intergenic regions, possibly related to unannotated transcripts. As in other studies, we saw a significant enrichment of RDDs in non-protein-coding DNA and regions enriched for Alu elements (Table S1 and Figure 2C).

We subsequently used two approaches to quantify the degree of RNA editing to help understand its role in AML. Using the *RNAEditingIndexer* tool,^19^ we estimated the Alu editing index (AEI), a global measure of RNA Editing for each sample. This measure has been widely used in associating altered editing patterns with patient survival probabilities across several cancers, and has helped in identifying global regulators of RNA editing.^8^^,^^11^^,^^12^ We demonstrated a positive correlation between ADAR1 expression and AEI across all patient samples (rho = 0.24, p value = 2.5e-03) (Figure 2D). However, we did not observe any association of the AEI with age or sex across the samples. The second approach involved site-specific analysis, where the degree of editing is measured by the ratio of A-to-G mismatched reads to the total number of reads mapped at that locus. Both approaches are used in several downstream analyses as shown in Figure 1.

### Acute myeloid leukemia demonstrates an altered degree of RNA editing

**Figure 3:**
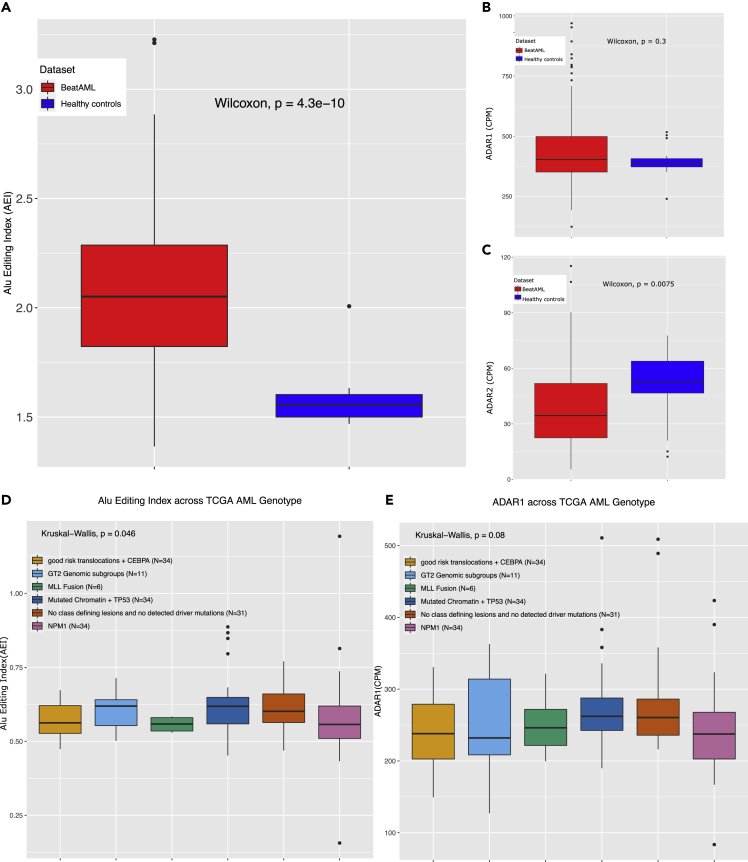
Differential editing in AML (A) Boxplots of Alu editing index (AEI) between AML patient samples (red) and healthy controls (blue) from BeatAML cohort. (B) Boxplots of ADAR1 gene expression (CPM) between AML patient samples (red) healthy controls (blue) from BeatAML cohort. (C) Boxplots of ADAR2 gene expression (CPM) between AML patient samples (red) healthy controls (blue) from BeatAML cohort. (D) Boxplot of Alu editing index (AEI) plotted against the samples categorized based on their mutation status from TCGA AML dataset. Number of patient samples in each group is shown in parenthesis (GT2 genomic subgroups - *greater than two genomic subgroups*). (E) Boxplot showing the differences in the ADAR1 expression levels (CPM) across TCGA AML genotypes. Number of patients in each group is shown in the parenthesis. Plotted are data points for each patient group, alongside the median, first and third quartile, and 95% confidence interval of median.

It has been widely reported that RNA editing is altered in several cancers.^11^^,^^12^ The majority of solid-organ tumors show an increase in editing in comparison to their counterpart normal tissue. As the TCGA AML lacked data from healthy individuals, we sought to look at differential patterns of RNA editing across a recently published AML dataset, the BeatAML cohort (Tyner et al., 2018), which includes transcriptomic data from 21 healthy samples. In line with other cancers, we also observed elevated levels of RNA editing in AML (Figure 3A). Increased levels of editing in several cancers have correlated with ADAR1 expression levels,^11^ however, ADAR1 is not differentially expressed between AMLs and healthy controls (Figure 3B). Interestingly ADAR2 is significantly downregulated in AML (Figure 3C). These differential expression patterns of ADAR1 and ADAR2 are further confirmed by a recent study exploring the functional role of RNA editing mediated by ADAR2 in the leukemogenesis in patients carrying t(8; 21) or inv16 mutations.^20^ However, unlike for ADAR1, no correlation was noted between ADAR2 levels and the AEI (Figure S1A)

### RNA editing varies according to acute myeloid leukemia genotype

Within the TCGA cohort, we further aimed to look at how RNA editing differs across different AML mutational genotypes. We hypothesized that the heterogeneity in RNA editing levels in AML may relate, at least in part, to the genetic heterogeneity evident within it. As previously documented, we observed genetic heterogeneity within our cohort and used an up-to-date disease classifier to subdivide AML into pathogenetic subtypes.^21^ However, for pragmatic reasons, due to the relatively small number of patient samples we collapsed together categories that are similar in terms of prognosis (see methods), to come up with six distinct groups that broadly represented the genetic heterogeneity of AML. Of note, we observed a statistically significant difference in the degree of editing across the different genetic categories (Kruskal-Wallis rank-sum test, p value = 0.046, Figure 3D). This observation suggests that the degree of RNA editing in AML differs according to the genetic sub-groups and infers a potential mechanistic link between mutation status and RNA editing. We further aimed to validate our findings of the TCGA cohort with the BeatAML dataset (Tyner et al., 2018). The numbers of patients in each mutational subgroup in both the TCGA AML and BeatAML datasets are of similar size with few exceptions (see methods). However, as in the TCGA AML cohort, we could further replicate significant differences in RNA editing levels, across AML genotypes within the BeatAML patient samples (p value = 0.036) (Figure S1B). As has been shown in an earlier study across AML cell lines and patient samples,^20^ we observed ADAR2 (Figure S1C), but not ADAR1 (Figure 3E), to be differentially expressed between different AML genotypes.

### Alu editing index correlates with survival in acute myeloid leukemia

**Figure 4:**
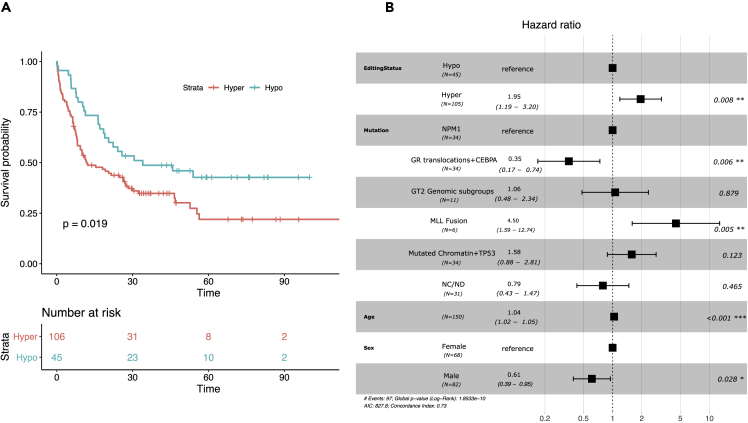
Increased levels of editing are associated to poor survival outcome (A) Kaplan-Meier analysis between hypo (AEI<0.546, N = 45) and hyper-edited samples from the TCGA AML dataset. Time in months is shown on x axis. p *value* is shown in the plot. (B) Multivariate survival analysis of TCGA AMLs using a cox proportional hazards model on editing status as a categorical variable. Mutation status, age, and sex are used as additional variables. Number of samples in each category is shown in parenthesis. p *values* and hazard ratios are shown in the plot (GR translocations + CEBPA - good risk translocations and CEBPA, GT2 genomic subgroups - *greater than two genomic subgroups*, NC/ND - *non class defining lesions and no detected driver mutations*).

We next sought to determine any link between RNA editing and survival, and, utilizing similar methodologies to previous studies (Figure S1D), we classified our patients into those “hypo” and “hyper”-edited. Sorting the AEI in ascending order, samples with the lowest 30^th^ percentile were considered to be hypo-edited (N = 45, AEI<0.546). Sample classification, including clinical data, is shown in Table S2. In keeping with reports in other cancers, Kaplan-Meier analysis demonstrated that hyper-edited patients have an inferior survival in comparison to hypo-edited patients (p value = 0.019, Figure 4A). Furthermore, we could replicate these findings across the BeatAML dataset, with elevated levels of editing associated with poor survival outcomes (p = 0.02) (Figure S1E).

To exclude an association of AEI with other prognostic variables within the TCGA data, we therefore extended this analysis by fitting a multivariate model using several clinical characteristics of known prognostic significance, such as mutation status, age and sex as additional variables.^22^ Prior to running the model, the proportional hazard assumption was approved using Schoenfeld residuals and graphical evaluation. Of note, hyper-edited patients continued to demonstrate a significantly worse prognosis in comparison to those hypo-edited (HR 1.9, CI 1.2-3.2, p value = 0.008 Figure 4B) in this multivariate analysis.

### Specific RNA editing events correlate with patient survival

**Figure 5:**
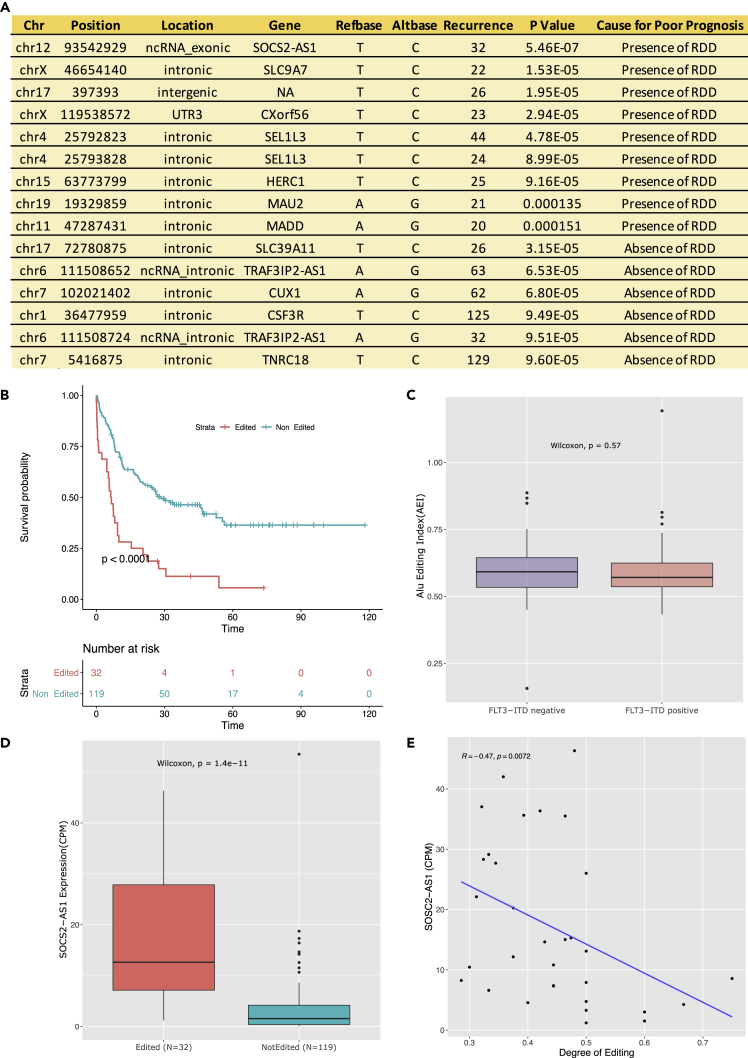
Association between presence/absence of RDD and survival outcome (A) Kaplan-Meier analysis between the samples with the presence and absence of an RDD in TCGA AML. Only the sites that are significant at FDR 20% (p *value* < 1.5e-04) are shown. (B) Kaplan-Meier analysis between the samples with (N = 32) and without an editing event (N = 119) of the editing site at SOCS2-AS1 at the loci chr12:935,429,429 (*T - > C*). (C) Boxplot showing the differences in Alu Editing Index (AEI) between the samples with and without *FLT3-ITD* mutation. Plotted are data points for each patient group, alongside the median, first and third quartile, and 95% confidence interval of median. (D) Boxplot showing the differences in the expression levels (CPM) of SOCS2-AS1 between the samples with (N = 32) and without an editing event (N = 119) within TCGA AML dataset. Plotted are data points for each patient group, alongside the median, first and third quartile, and 95% confidence interval of median. (E) Correlation between SOCS2-AS1 expression (CPM) and the degree of RNA editing for 32 edited patient samples.

To try and correlate patient survival with specific RNA editing events, we next performed survival analyses, plotting patient Kaplan-Meier curves for every RDD site, based on their editing status. To provide adequate statistical power, we chose sites where at least 20 patients could be identified that either demonstrated or lacked an editing event (N = 19,691). At FDR 20% (p value <1.5e-04), fifteen editing events, predominantly located in non-protein-coding DNA, conferred a markedly poorer survival outcome for those patients who either demonstrated or lacked an editing event (Figure 5A). The most statistically significant editing site, predictive of a poorer outcome (p value = 5.4e-07, Figure 5B), was recurrent in 32 patients (chr12: 93,542,929, T>C) and was located in an exonic region of a non-protein-coding RNA (*SOCS2-AS1*). It has recently been reported that *SOCS2-AS1* modulates *FLT3-ITD* activity via sponging *miRNA-221* activity to maintain STAT5 signal transduction.^23^ However, we observed no significant differences in *FLT3* mutational status between the number of patients with (11/32 cases, 34%) and those that lacked an RDD (33/119, 28%). Moreover, the AEI also did not alter between patients that carried or lacked the *FLT3-ITD* mutation (Figure 5C). Multivariate survival analysis was performed to estimate the effect of the *FLT3* mutation on the overall survival of the patients with the editing event and observed that poor outcome appears to be independent of the *FLT3* mutation status (Figure S1F). However, as STAT5 can be activated by other pathways in AML^24^ we cannot rule out STAT5 activation as the potential link.

Of interest, edited patients showed a markedly increased expression of *SOCS2-AS1* in comparison to non-edited patients (N = 119) (Figure 5D). We next checked if the degree of editing was in any way correlated with gene expression. To our surprise, within the patients with edited *SOCS2-AS1*, we observed a strong negative correlation between the degree of editing and the mRNA abundance (Figure 5E). Taken together, these data demonstrate that the editing site at *SOCS2-AS1* is correlated with poor survival and acts as a potential regulatory editing event with higher expression levels in edited patients but demonstrates an inverse correlation between the degree of editing and mRNA abundance.

### Regulatory RNA editing events

**Figure 6:**
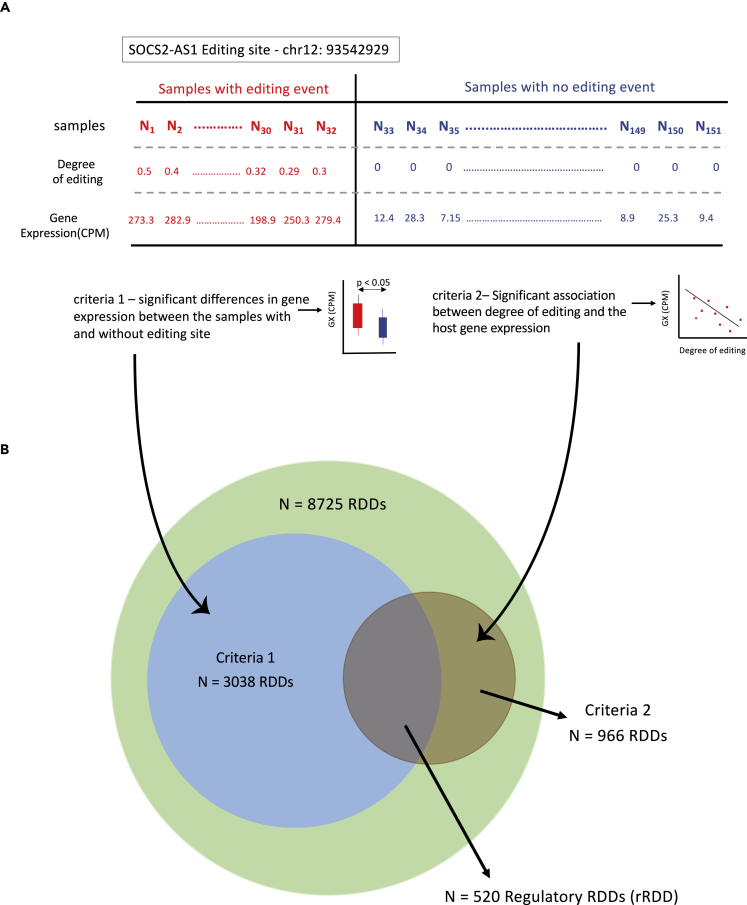
Regulatory RDDs (A) Across all the RDDs from the TCGA AML dataset, samples are dichotomized based on their editing status. Samples with and without an editing site are colored in red and blue respectively. Regulatory RNA-DNA differences (rRDD) are identified based on two criteria. 1) Significant differential gene expression between the samples that have or lack an RDD. 2) Significant correlation between the degree of editing and the host gene expression, irrespective of whether these genes satisfy criteria 1. (B) Overlap of criteria 1 and 2 (Figure 6A) showing the number of rRDDs.

Building on the potential regulatory effect of the presence of an editing event on *SOCS2-AS1* expression levels, we next analyzed whether this was also a general phenomenon across all RDDs (STAR methods and Figure 6A). We sought to systematically determine how expression patterns for linked genes differ between patients that demonstrated or lacked RDDs. To allow for stringent statistical comparison, we limited this analysis to RDDs that are recurrent in at least 30 individuals. This led to the identification of 8725 RDDs that directly mapped to 2332 genes (Table S3). At every RDD we compared gene expression levels between the samples, based on their editing status. At an FDR of 1% (p value <0.01), patients with an editing event expressed the gene at higher levels in comparison to patients that lacked an RDD at more than one-third of these RDD sites 3038/8725 (35%) (Figure 6B).

We further extended our analysis to determine how the degree of editing, rather than its binary presence or absence, correlated with the expression of the primary transcript. From 8725 possible associations, at FDR <10% (p value < 0.018) we found 966 sites (966/8725, 11%) corresponding to 610 genes (610/2332, 26%), where the degree of editing significantly correlated with the expression level of their transcript (Figure 6B). Remarkably, almost all of the sites (N = 938, 97%) were negatively correlated with host gene expression (Table S3), replicating the pattern at *SOCS2-AS1*. We estimated 520 RDDs mapping to 337 genes overlapping from the above two analyses, i.e. where the edited patients demonstrate higher levels of gene expression than the unedited patients, but the degree of editing negatively correlated with the transcript level (Figure 6B). We have suggested these as regulatory RNA-DNA differences (or rRDD) that could potentially act as *cis-regulatory* variants to determine mRNA levels (Table S3).

To understand the functional relevance of the genes that are associated with rRDDs, we then sought to estimate the extent of their overlap with genes differentially expressed between blasts from patients with AML and from healthy individuals (see methods), to determine if this difference might relate to their edited status. Of note, we found that 33 of 337 genes (10%, p < 0.003) linked to rRDD, overlapped with genes up-regulated in the BeatAML dataset (Table S3), but that negligible overlap (<1%) with downregulated genes was observed. Importantly, these observations suggest that altered RNA editing may also contribute, at least in some part, to the gene expression differences evident between AML and normal hematopoeitic tissue.

### Genetic variants regulating RNA editing

Quantitative trait loci (QTLs)-mapping is an approach that has been widely used to find an association between genetic variants and phenotypic variation, such as gene expression (eQTL),^25^ splicing (sQTL)^26^ and DNase1 sensitivity (dsQTL).^27^ In line with this, to understand potential factors regulating the degree of RNA editing, we performed edQTL (editing quantitative trait locus) analysis by associating the common genomic variants (minor allele frequency MAF>0.05) that are located 100 kb on either side of the RNA editing site.^28^^,^^29^ To this end, edQTL analyses were performed with approximately two million genotypes that were imputed against the 1000 genomes reference panel.^30^ Editing sites that are recurrent in at least 40% (3590 editing sites) of the individuals were used for this analysis. Missing editing values were imputed using the missMDA package.^31^ Taking the genetic variants in close proximity (±100kb window on either side of the RDD), we performed 451,285 association tests. At FDR 20% (p value = 2.3e-03), 4873 genetic variants were found to have a significant association with 200 editing sites that are mapped to 163 genes (Table S4). Hereafter, these RNA editing-associated SNPs are referred to as edSNPs. There is an enrichment of associations with very low p values (Figure S2A). Similar to the distribution of editing sites across the genome, we see an enrichment of these edQTLs in intronic and 3′ UTRs; however, the corresponding edSNPs are largely located in intronic and intergenic regions (Figure S2B).

We next sought to understand if there is any shared or coordinated genetic regulation of RNA editing with the expression of the corresponding gene. For this, we performed gene expression QTL (eQTL) analysis from the same dataset and overlapped variants of interest with our edQTLs identified above. As reported in several studies, we performed eQTL association analyses with the same imputed genotypes within a 1 MB window from the TSS of the genes that are expressed in all the samples (N = 13,166). At FDR 10% (2.87e-04), we found 3274 genes to have a significant association with a nearby genomic variant. However, when the linkage disequilibrium (LD) structure (R^2^ > 0.8) of the genomic variants was taken into account, we found only five genes (*ABCB10, D2HGDH, MCM3AP, POLR2D, and SENP5*) (Figure S3) to have a common variant associated to the degree of RNA editing and to the expression of the corresponding gene, effectively showing the independence of edQTLs and eQTLs.

### Functional importance of editing quantitative trait locus associations

Upon testing 3590 RDDs, we found 200 edQTLs to have an association with the nearby genetic variant (edSNPs). To determine the potential functional relevance of these associations, we sought to determine the extent of overlap of the rRDD. We found 27 (13.5%) of the 200 edQTLs (Table S5) to overlap with the rRDDs.

We noted that the majority of edSNPs are located in non-protein-coding DNA (Figure S2B). Multiple genome-wide studies have determined that non-protein-coding DNA harbors not only disease-susceptible loci but contains several functional elements that regulate the genome.^32^^,^^33^^,^^34^ It has been reported that the majority of DNase hypersensitive sites (DHSs), known to be enriched for such regulatory elements, are located in the intronic and intergenic regions.^35^ We therefore analyzed our edSNPs with DHSs using the recently developed FORGE2 method (https://forge2.altiusinstitute.org/) which detects tissue and cell-type specific enrichment in epigenomic regions including DNase I hotspots, histone mark broadPeaks and Hidden Markov Model (HMM) chromatin states.^36^ Interestingly, when applying FORGE2 analysis across different tissues, we found that our edSNPs are enriched in blood-related cell lines including those from the human leukemia cell line HL-60, derived from an AML patient (Figure S2C). Another recent resource from ENCODE (https://screen.encodeproject.org/) integrated DHS regions with multiple epigenetic signals, namely H3K4me3, H3K27ac, and CTCF ChIP-Seq and defined candidate Cis-Regulatory elements (cCREs) across different cell lines.^37^ To estimate if the overlap of our edSNPs with cCREs is expected more than by chance, we applied the permutation approach with 1000 iterations (see methods). As expected, we observed obvious tissue-specific enrichment with the highest overlap observed for hematopoeitic tissue and particularly hematopoietic malignancies (23/30 top Z-scores, Figure S2D). Further classification of cCREs epigenetic modification signals within the exemplar HL-60 cell line revealed over representation of edSNPs across regions with high DNase-seq and H3K4Me3 signals (Figure S2E), suggesting that the edSNPs reside within classical cis-regulatory elements.

## Discussion

A-to-I RNA editing is a post transcriptional modification that is ubiquitous across Metazoans.^38^ Abnormal RNA editing levels at coding or regulatory RNA sequences are now acknowledged to make a significant contribution to several cancers.^9^^,^^11^^,^^39^ However, although the role of RNA editing in AML was first described almost two decades ago, previous studies have been limited in size and its role remains undetermined. Using the TCGA AML dataset, here we studied the functional and regulatory role of RNA editing in AML linking this to patient characteristics including survival, cytogenetic and mutational status. We further demonstrate that RNA editing is aberrant in AML, associate editing to RNA abundance, and identify genetic variants linked to the degree of editing in AML.

More recently, global measures of RNA editing were estimated in patient cohorts from the TCGA and demonstrated elevated levels of editing across multiple primary solid-organ cancers. These differences in AEI are largely explained by ADAR1 expression levels.^11^ In agreement with other cancers, we observed elevated levels of editing levels in AMLs, however this does not correlate with differential ADAR1 expression (Figures 3A and 3B).

We further observed significant differences in the editing levels across patient samples with different AML genotypes. Of note, in general, the genotype groups with low risk, including good risk translocations, *CEBPA,* and *NPM1* mutations, appear to have a lower degree of RNA editing in comparison with patients carrying high-risk mutations (i.e. patients with mutated chromatin regulators and *TP53*). The AEI index is positively correlated with ADAR1; however, we observed no significant differences in ADAR1 expression levels across the AML genotype (Figure 3E, p *value* = 0.08). A recent study reported that ADAR2 is significantly downregulated in patients with t(8; 21) or Inv16 mutations, carrying *RUNX1-ETO* and *CBFβ-MYH11* fusion proteins. Tenen and colleagues have experimentally demonstrated *RUNX1-ETO* repression of ADAR2 expression and proposed a mechanism whereby hypo-editing of specific transcripts by ADAR2 was implicated in the pathogenesis of t(8; 21) AMLs.^20^ We did not observe a general correlation of ADAR2 with the AEI (Figure S1A), but did observe a distinct pattern of associations between ADAR1 and ADAR2 with AEI across each AML mutation subtypes (Figure S1G) suggesting individual interactions of ADAR enzymes with specific mutations in AML subgroups. Further studies are warranted to identify the potential interactions of ADARs with disease-causing mutations.

Previous studies in breast invasive carcinoma (BRCA), liver hepatocellular carcinoma (LIHC), head and neck squamous cell carcinoma (HNSC) and glioblastoma multiforme (GBM), have demonstrated that the AEI correlates with overall survival^11^^,^^12^ (Figure S1D). To determine if such an association existed for AML, we performed survival outcome analysis and indeed demonstrated hyper-edited AML patients to have a poor prognosis. An obvious explanation for this would be our discovery that the degree of editing relates to the specific AML genotype, and that poor-risk characteristics are associated with a greater degree of editing (Figure S2F). However, the continued association of editing with a poor prognosis in multivariate analysis suggests an effect independent of genotype.

We further assessed the potential effects of each individual editing site on the patient’s survival outcome. Interestingly, we found that the presence of an RDD in the exonic location of the ncRNA *SOCS2-AS1* (chr12: 93,542,929, T>C) predicted a poor outcome. In addition to RDD at the *SOCS2-AS1* locus, we found many sites that demonstrated an association between the degree of editing and steady-state mRNA levels*.* However, this relationship appears complex given that the presence of an editing event was often associated with a higher steady state mRNA for the primary transcript, but the degree of editing inversely correlated with the mRNA abundance. Although initially counterintuitive, this suggests that RNA editing may be an additional layer of control to regulate transcript abundance, and perhaps particularly in some highly abundant transcripts, although whether this editing alters RNA stability or turnover remain mechanistically elusive and warrants further investigation. Moreover, the significant overlap of these genes with those overexpressed in AML vs healthy controls suggests that, along with several genetic and epigenetic factors, RNA editing in AML may contribute to disordered transcription and the malignant phenotype.

In summary, we present the first large-scale RNA editing analysis from AML patient samples. Our results demonstrate that increased levels of editing are associated with poor prognosis, that the AEI varies across AML genotypes and that there is an association between the degree of RNA editing and expression levels of multiple genes, some of which are dysregulated in AML in comparison to normal hematopoiesis . Our edQTL analysis revealed that one of the factors potentially influencing the altered levels of editing are *Cis-regulatory* variants. Earlier studies have shown that several RNA binding proteins (RBPs) facilitate editing by interacting with ADARs or through binding to Alu elements in a tissue-specific manner.^40^ Using K562 and HepG2 cell lines, it has been shown that both Transcription Factors (TFs) and RBPs co-occupy functional hotspots on the genome in a coordinated manner, highlighting the fact that both transcriptional and co-transcriptional mechanisms are more functionally interconnected. Encode data has further suggested that RNA binding proteins (RBPs) preferentially bind to open chromatin regions and DNase1 hypersensitive sites.^41^ As we found our edSNPs to enriched at these regions, we speculate that the binding of TFs or other RBPs may play a crucial role in regulating editing levels within the primary transcript, perhaps through 3D-communication and suggest that this hypothesis should initiate further investigation.

### Limitations of the study

Our study aims to understand the potential role of RNA editing in the pathogenesis of AML; however, it has some limitations. To enable us to study RNA Editing, we have accessed the most comprehensive transcriptomic data of patients with AML from the TCGA, a dataset that also benefits from having cytogenetic data regarding mutational events and clinical outcome data, allowing us to further correlate RNA editing with these disease characteristics. However, an obvious drawback of this data is that it lacks a matched control dataset, both at the level of the individual patient and an appropriate cell type. Therefore, to study the global differences between AML and healthy controls we utilized transcriptomic data from a validating external dataset, the BeatAML dataset. Within the BeatAML cohort, we validated our findings by observing AMLs to have an elevated degree of RNA editing in comparison to healthy controls.

Although our cohort is the largest with transcriptome, genome, and clinical data available, another limitation of our study is the cohort sample size, which is relatively modest at 151 patients, with another 135 in the validating dataset. However, AML is a clinically heterogeneous disease that is characterized by a large number of recurrent gene mutations and chromosomal abnormalities. A recent panel-based genome study, performed on 1540 patients, classified AML into *fourteen* individual sub-groups. Given our relatively small dataset of 151 patients, to allow us any statistical power, we therefore had to rationalize this classification system, collapsing similar biological and clinical groups together into a more manageable seven genetically similar groups. Decades of genetic research have taught us that increasing the sample size can add significant mechanistic granularity, therefore our underpowered study may have underestimated or missed associations between RNA editing and AML biology. However, despite our limited cohort we were still able to (i) demonstrate variable RNA editing across AML genotypes, (ii) describe that higher levels of editing are a predictor of poor outcome, (iii) observed elevated degree of editing in AMLs in comparison to healthy individuals, (iv) demonstrate that RNA editing is one of the contributors to altered gene expression in AML and (v) have identified edQTL to identify putative cis-regulatory regions that regulate RNA editing, and could validate the clinically relevant findings i-iii) in an independent AML cohort, the BeatAML dataset.

## STAR★Methods

### Key resources table

**Table:** 

REAGENT or RESOURCE	SOURCE	IDENTIFIER
**Software and algorithms**		

GATK	McKenna et al.^42^	https://gatk.broadinstitute.org/hc/en-us
Annovar	Wang et al.^43^	https://annovar.openbioinformatics.org/en/latest/
*RNAEditingIndexer*	Roth et al.^19^	https://github.com/a2iEditing/RNAEditingIndexer
BamUtil	Jun et al.^44^	https://github.com/mskilab/bamUtils
Survival	Therneau and Grambsch^22^	https://github.com/therneau/survival
Survminer	NA	https://rpkgs.datanovia.com/survminer/index.html
edgeR	Robinson et al.^45^	https://bioconductor.org/packages/release/bioc/html/edgeR.html
DESeq2	Love et al.^46^	https://bioconductor.org/packages/release/bioc/html/DESeq2.html
QTLtools	Delaneau et al.^47^	https://qtltools.github.io/qtltools/
missMDA	Josse and Husson^31^	http://factominer.free.fr/missMDA/index.html
GTC2VCF	NA	https://github.com/freeseek/gtc2vcf

### Resource availability

#### Lead contact

Further information and requests should be directed to the lead contact, Eshwar Meduri (em540@cam.ac.uk).

#### Materials availability

This study did not generate new unique reagents.

### Method details

#### Reads processing and calling RNA editing events

To estimate the RNA Editing events in the TCGA AML, we obtained the transcriptomic reads of 151 patient samples from the NCI GDC portal.^48^ Reads were aligned to the hg38 reference genome and uniquely mapped reads were retained and PCR duplicates were removed using PICARD tools. Filtered reads were further processed through RNA-Seq best practices pipeline in GATK, version 4.0.2.1 which includes Split’N’Trim, indel realignment and base recalibration.^42^ Variant calling was performed using HaplotypeCaller algorithm with parameters dontusesoftclippedbases and standcallconf = 20. Following this, SNP calls were filtered using GATK-VariantFilteration with clusters of at least 3 SNPs that are within a window of 35 bases, Fisher Strand values >30.0 and Qual By Depth values <2.0.

As all the patient samples do not have the corresponding whole genome sequence available, RNA editing events were quantified by overlapping with the published datasets.^8^^,^^9^ To avoid false positives, SNPs were further filtered from the genomic variants listed in the 1000 genomes project (dbSNP build 151).^49^ We discarded the reads with mismatches at the 5′ end.^50^ As described earlier, we quantified RNA editing sites with at least three mismatches and one mismatch at non-Alu and Alu sites respectively.^8^ Using a very conservation approach, we finalised the RNA editing events that are recurrent in at least five individuals. At each editing site we calculated the degree of editing by dividing the reads with altered bases by the total number of reads. RDDs are annotated using ANNOVAR.^43^

For replication purposes, we obtained transcriptomic data of AML patient samples from BeatAML cohort.^16^^,^^48^ Reads were processed using the same pipeline as TCGA AML dataset. Samples from BeatAML contains a mixture of samples collected from bone marrow and peripheral blood. Interestingly we found significant differences of RNA editing levels between the sample types (Figure S2G). For all replication analysis we retained only the samples from peripheral blood to match the data to that of TCGA AML. In addition, a few samples were sequenced more than once, probably due to relapse of disease. However, as the timing of the samples and the clinical condition of the individual at that time were not given, due our lack of knowledge of the potential evolution of RNA-editing with disease progression, these samples were also removed from subsequent analysis. This resulted in 135 AML patient samples from the BeatAML cohort.

#### Global measurement of the degree of editing

The majority of Adenosines genome-wide have variable reads mapped and are edited at a very low level (<1%). With the trade-off between the sequencing costs and the number of reads, it is not feasible to capture editing levels of all Adenosines in large cohorts. To overcome this, a metric called the Alu Editing Index (AEI) was designed to measure global editing levels in each sample.^19^^,^^51^ It has been reported that estimation of AEI is sensitive to read lengths and the alignment process. To maintain uniformity, we realigned the reads from both the datasets with the same parameters. To match with read lengths of TCGA AML patient samples we trimmed the reads of the BeatAML samples to 50 bp using BamUtil package^44^ before estimating AEI.

#### Regrouping of AML patient samples

Based on a recent study performed on 1540 patients, AML can be classified into 14 sub-groups.^21^ However, due to our relatively small sample size in the TCGA AML and to the lack of or small number of patients in some of these groups, in an attempt to obtain statistical significance, we sought to rationalise this classification by amalgamating genetically similar groups. Using this practical approach, we came up with seven distinct groups as shown in Table S2. Patients with the IDH2^R172^ mutations sub groups contained only one patient, that was excluded, whereas patients with NPM1, mutated chromatin+TP53, good risk translocations+CEBPA, > 2 genomic subgroup eligibility and no class defining lesions+driver mutations, were present at a range of 6–34 patients (see Table S2). We used a similar approach to group the BeatAML patients. Number of patients in each mutational subgroup in both the TCGA and the BeatAML are of similar size with a few exceptions. The “GT2 Genomic subgroups (greater than two genomic subgroups)” patient group is missing in the BeatAML and the size of the “Mutated Chromatin+TP53” subgroup in the BeatAML dataset is proportionally larger than in the TCGA dataset (Table S2).

#### Statistical analysis

All the statistical analyses were performed using the R statistical package and figures were plotted using the ggplot2 package. Kaplan Meier, univariate and multivariate survival analyses were performed using the “survival”, “suvminer” and “dplyr” packages.

#### Gene expression analysis

RNA-Seq counts for each gene were processed with HTSeq package.^52^ Read counts were normalised and CPM (counts per million reads) values were estimated using edgeR package.^45^ Differential expression analysis was carried out using Bioconductor package DESeq2.^46^ Genes are considered to be differentially expressed at 5% Bonferroni correction and log fold change of ±1.5.

#### Estimating regulatory editing sites

To establish the correlation between the degree of editing and gene expression, we used a two-step approach. First, we shortlisted the editing sites with differential mRNA abundance within the same gene between edited and non-edited samples. A simple Wilcoxon test was applied on normalised CPM values. In the second step, among the edited samples, significant correlations should exist between the degree of editing and host gene expression. For this, we applied a multiple regression model to check for an association between the gene expression levels and the degree of editing, taking age and sex as covariates (ΔGX ∼ ΔdRDD + sex + age). Regulatory editing events are quantified by overlapping filtered significant events fulfilling both the above criteria. As an exemplar, we chose RDD at chr12: 935429492 in *SOCS2-AS1* gene (Figure 6A).

#### Genotype imputation

All the patient samples were genotyped on the Affymetrix Genome-wide Human SNP Array 6.0. Raw data was downloaded from GDC data portal (https://portal.gdc.cancer.gov/) and was processed using Affymetrix library files and converted to the VCF format using https://github.com/freeseek/gtc2vcf. Prior to imputation we followed several QC steps as follows 1) we filtered for common variants (MAF > 0.01) and deviations from HWE (1e-05). 2) Two samples were removed after checking for high levels of missing data. Data was pruned at LD > 0.2. 3) We excluded high LD and non-autosomal regions from the pruned file. 4) Related individuals (IBD, identical-by-descent >0.1875) were further discarded.^53^ Comprehensive genotype imputation was run on the Sanger imputation server against the 1000 genomes reference panel using pre-phasing and imputation with EAGLE2+PBWT pipeline.^30^ After imputation, SNPs were filtered at a MAF > 5% and info value of >0.8. As a sanity check, we validated the imputed genotypes with the sequencing data Match BAM to VCF (MBV).^54^

#### edQTL analyses

Associations between the degree of editing (dRDD) and the imputed genotypes were performed using QTLTools package.^47^ We filtered samples by matching them with those of imputed genotypes and created a matrix with the editing levels. Editing sites that are recurrent in at least 40% of the samples (N>59) were used for this analysis. Missing values in the matrix are imputed using missMDA package.^31^ PCA was performed on the dRDD and the genotype files using QTLTools. As the imputed genotypes are mapped to hg19, we converted the location of the editing sites from hg38 to hg19 using liftOver. Taking age, sex, five genotype PCs and two editing PCs as covariates, we performed a *Cis* nominal analysis limiting to SNPs located 100kb on the either side of the editing location. False discovery rate was calculated using qvalue package in R.^55^

#### eQTL analyses

As described in the GTEx consortium eQTL analysis pipeline, read counts were normalised using trimmed mean of M values (TMM).^56^ Prior to that, genes were filtered based on the criteria 1) ≥ 0.1 TPM in ≥ 20% samples and 2) ≥ 6 unnormalised reads in ≥ 20% samples GTEx.^57^ Using QTLTools package we performed eQTL analysis with the imputed genotypes using 1MB window against the normalised gene expression values.^47^ Age, sex, five genotype based principal components and 30 PEER factors were used as covariates for eQTL mapping.

#### FORGE2 and cCRE analysis

FORGE2 command line analysis was performed on 4,305 top edSNPs using standard settings and ENCODE data (1000 background repetitions, ENCODE DNase I hotspot data analysis setting.^36^ For cCRE analysis we downloaded cCREs regions across all the cell lines that are reported in the SCREEN database. They are classified based on the occupancy of DNase, H3K4me3, H3K27ac and CTCF signals. Regions with low-DNase and Unclassified (low H3K4me3, H3K27ac or CTCF) were excluded. To test for the enrichment of edSNPs across cCREs, a permutation test was performed with 1000 iterations using the regioneR package.^58^

### Quantification and statistical analysis

We used the Wilcoxon test for comparisons between two groups and the Kruskal–Wallis test for comparisons between more than two groups. To compare two continuous variables we used Pearsons correlation method but with additional covariates we applied multiple regression model. Kaplan-Meier analysis was used to estimate survival outcomes and hazard ratios were calculated using a multivariate Cox proportional hazards regression model. The *qvalue* package was utilized to adjust the p values during multiple testing. Unless otherwise stated, all the statistical tests were considered to be significant with a p value < 0.05. All the statistical tests were performed using the R package.

#### Availability of data and materials

The data used for the analyses described in this manuscript were obtained from the dbGaP website under accession number TCGA: phs000178.v11.p8 and BeatAML: phs001657.v2.p1. All the data generated is available along with this article.

## Data Availability

• All data reported in this paper are shared as supplementary data. • This paper does not report original code. • Any additional information required to reanalyse the data reported in this paper is available from the lead contact upon request.

## Supplemental information

Document S1. Figures S1–S3

Table S1. List of RDDs in AML A) List of RDDs in TCGA-AML. B) List of exonic RDDs. C) Overlap of AML RDDs with Han.et al. 2015, related to STAR Methods and Figure 2.

Table S2. Clinical classification of patients with AML A) Summary of TCGA AML samples grouping based on their mutation status. B) Hypo/Hyper AEI classification and clinical information of TCGA AML patient samples. C) Hypo/Hyper AEI classification and clinical information of BeatAML patient samples, related to STAR Methods and Figure S4.

Table S3. Regulatory RDDs A) List of RDDs recurrent in atleast 30 AML patient samples. B) Differential gene expression analysis between samples with and without an RDD. C) multiple regression analysis between degrees of editing and host gene expression levels taking age and sex as covariates (ΔGX ∼ ΔdRDD + Sex + Age). Regression coefficient and *p-values* are shown. D) List of Regulatory RNA-DNA differences (rRDD). E) Genes mapped to rRDDs. F) List of overlapping genes associated with rRDDs and upregulated genes in AML, related to STAR Methods and Figure 6.

Table S4. edQTL association A) List of top edQTL association (based on *p value*) of RDDs with a genetic variant within 100kb window. B) List of all significant edQTL associations of RDDs with a genetic variant within 100kb window, related to STAR Methods.

Table S5. Overlap of edQTLs with rRDD loci, related to STAR Methods
