# Supplementary material for: The RNA editing landscape in acute myeloid leukemia reveals associations with disease mutations and clinical outcome
Source: iScience. 2022 Nov 17;25(12):105622. doi: 10.1016/j.isci.2022.105622 (PMC9713371; doi:10.1016/j.isci.2022.105622)
Supplement: Document S1. Figures S1–S3 [file mmc1.pdf]

iScience, Volume *25*

Supplemental information

The RNA editing landscape in acute myeloid leukemia reveals associations with disease mutations and clinical outcome

Eshwar Meduri, Charles Breeze, Ludovica Marando, Simon E. Richardson, and Brian J.P. Huntly

Supplementary figures and legends

Supplementary Figure S1

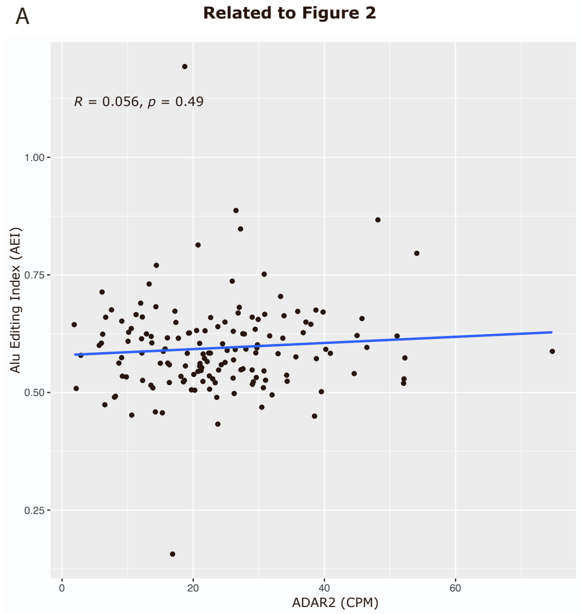

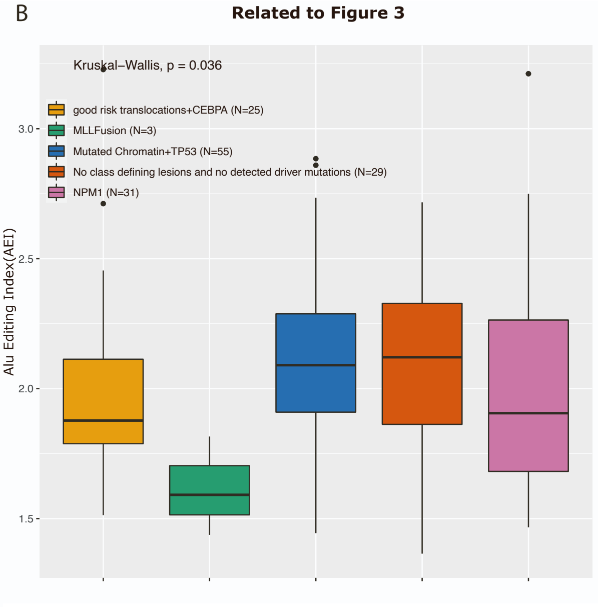

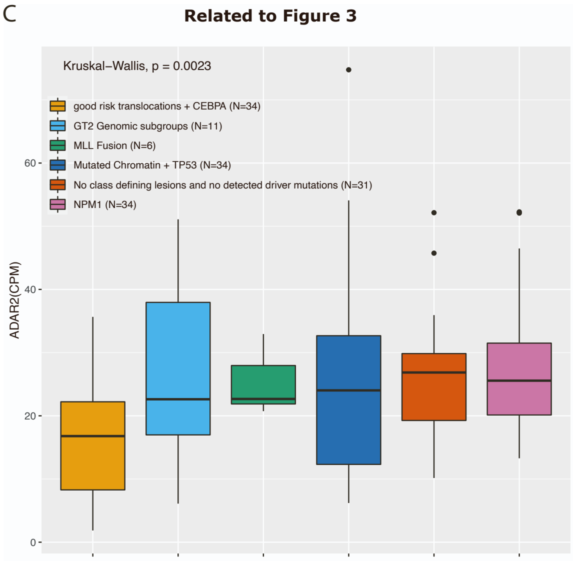

D

Related to STAR Methods

Cancer	AEI cutoff	pvalue	Article
BRCA	0.51	0.045	Paz-Yaacov et al 2015
HNSC	0.37	0.00075	Paz-Yaacov et al 2015
LIHC	0.69	0.0012	Paz-Yaacov et al 2015
GBM (Male)	0.78	0.032	Silvestris et al 2019
GBM(Female)	0.8	0.022	Silvestris et al 2019

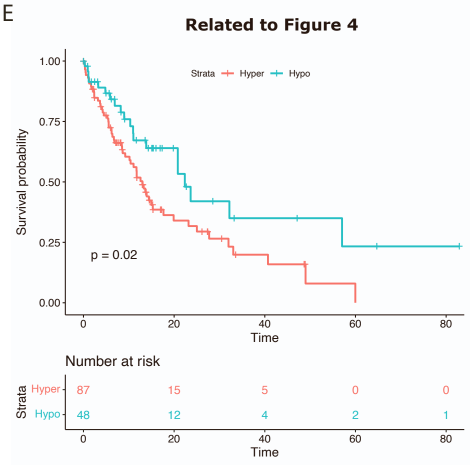

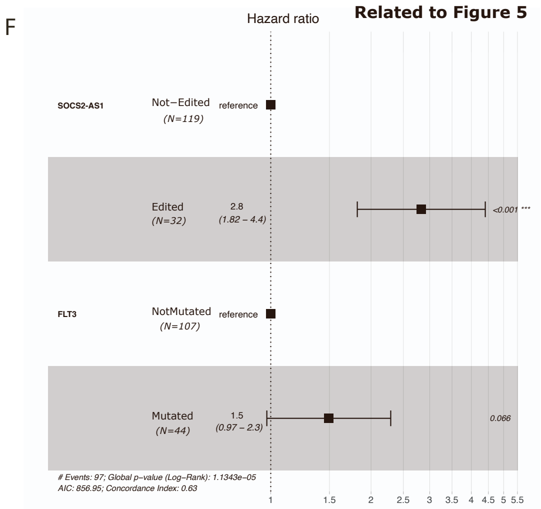

G

Related to STAR Methods

Patient subgroup	ADAR1	ADAR2
NPM1	0.41*	NS
GR translocations+CEBPA	NS	NS
Mutated chromatin+TP53	NS	NS
NC/ND	0.49**	NS
GT2 genomic subgroups	NS	NS

Figure S1 - Differential editing and association with clinical outcome. A) Correlation plot between AEI and ADAR2 gene expression (CPM) across TCGA AML. Each dot represents an AML patient sample. The straight blue line represents the linear relationship between the AEI and ADAR2 expression. B) Box plot of Alu editing index (AEI) plotted against the samples categorised based on their mutation status from BeatAML dataset. Number of patient samples in each group are shown in parenthesis. Plotted are data points for each patient group, alongside the median, first and third quartile, and 95% confidence interval of median. C) Boxplotsshowing the differences in the ADAR2 expression levels (CPM) across TCGA AML genotypes. Number of patients in each group are shown in the parenthesis. Plotted are data points for each patient group, alongside the median, first and third quartile, and 95% confidence interval of median. D) Studies showing AEI as a prognostic predictor in several cancers (Breast invasive carcinoma - BRCA, liver hepatocellular carcinoma - LIHC, head and neck squamous cell carcinoma - HNSC, glioblastoma multiforme - GBM). E) Kaplan-Meier analysis between hypo (AEI<1.889, N=48) and hyper edited samples from the BeatAML dataset. Time in months is shown on x-axis. *p-value* is shown in the plot. F) Multivariate survival analysis between hypo and hyper edited patients taking FLT3-ITD mutation status as an additional variable. G) Correlations between ADAR enzymes (ADAR1 and ADAR2) and AEI within each patient subgroup from the TCGA AML cohort. Patients carrying MLL fusion were not included as the group size is small (N=6). **p-value*=0.01, ** *p-value*=0.004, NS: not significant

Supplementary Figure S2 (Related to STAR Methods)

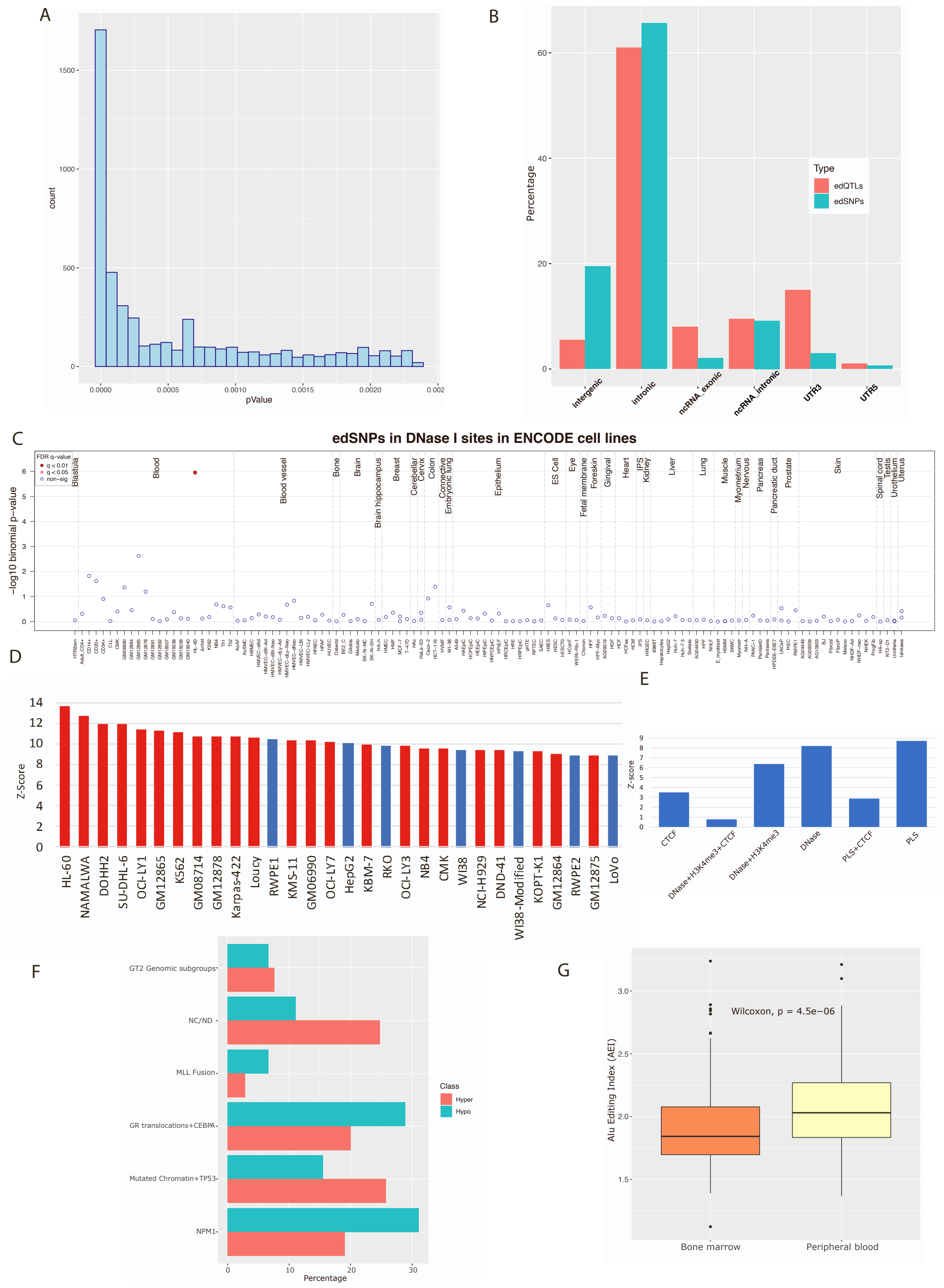

Figure S2 - edQTL Analysis. A) Histogram showing the frequency of significant p values of TCGA AML edQTL associations B) Distribution of edQTLs and edSNPs genomic locations from TCGA AML dataset C) Forge analysis from TCGA AML edSNPs in Dnase1 regions across different Encode cell lines. Cell lines are shown on the x-axis and the y-axis represents -log(10) of the binomial p-value. The highly significant HL-60 cell line is highlighted in the red circle. D) Z-scores for the significance of overlap of edSNPs with cCREs elements across different cell lines from the SCREEN database (https://screen.wenglab.org/). Top 30 sorted on z-score are shown in the plot. Haematopoietic cell lines (23/30) are highlighted in red. E) Individual classification of each category of cis-regulatory region in the HL-60 cell line. PLS - promoter like signatures fall within 200bp of TSS with high DNase and H4K4me3 signals, CTCF- CTCF bound, H3K4me3 - H3K4me3, DNase- DNase sensitivity site. Enrichment z-scores on *y-axis* are calculated using regioneR package using 1000 permuations. F) Percentage (shown on x-axis) of hypo and hyper edited samples across different AML genotypes (shown on y-axis) (GR translocations + CEBPA - good risk translocations and CEBPA, GT2 genomic subgroups - *greater than two genomic subgroups*, NC/ND - *non class defining lesions and no detected driver mutations*). G) Within the BeatAML patient samples, boxplots showing the differences of Alu editing index (AEI) between the samples collected from peripheral blood and bone marrow. Plotted are data points for each patient group, alongside the median, first and third quartile, and 95% confidence interval of median.

Supplementary Figure S3 (Related to STAR Methods)

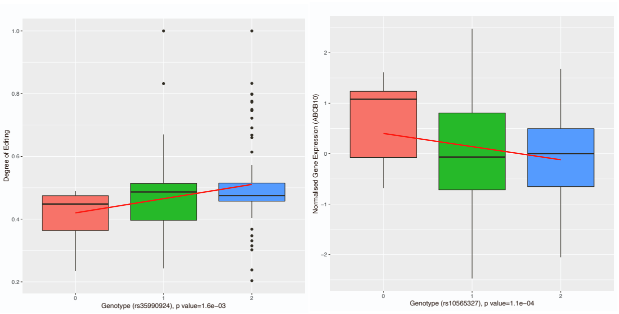

ABCB10

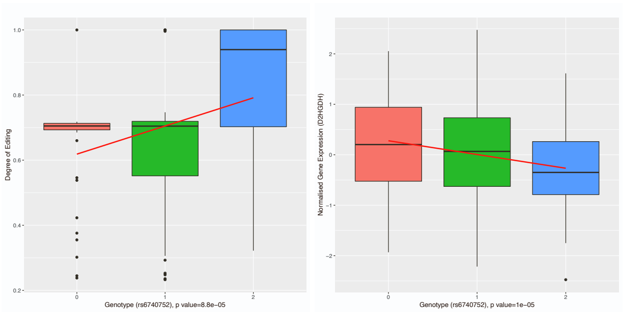

D2HGDH

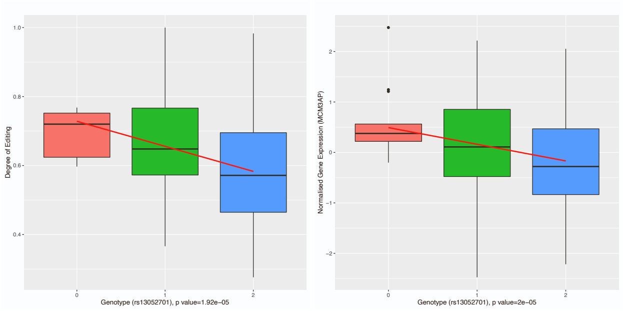

MCM3AP

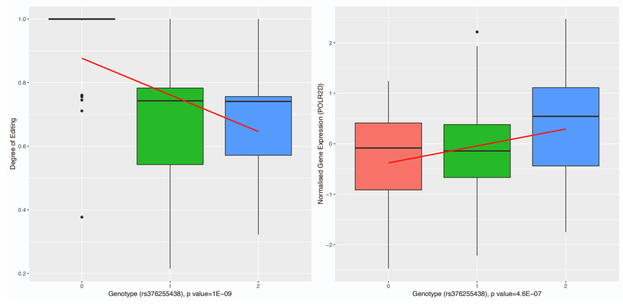

POLR2D

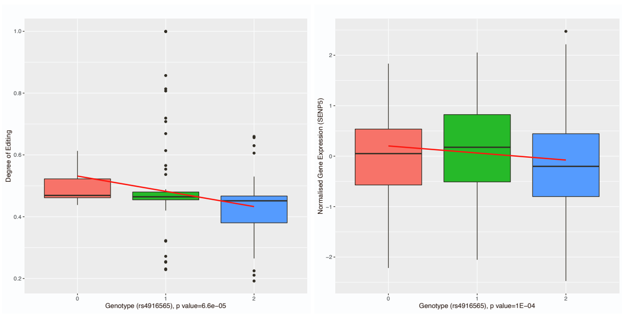

SENP5

Figure S3 – Overlap of edQTL and eQTL. Relationship between genotype and degree of editing (left panel) and gene expression (right) for the common variants associated to the degree of RNA editing and to expression of the corresponding gene.
